# Clinical applications of telemedicine services using a regional telemedicine platform for cancer treatment: a cross-sectional study

**DOI:** 10.1186/s12885-024-12563-5

**Published:** 2024-07-07

**Authors:** Fangfang Cui, Xu Zhang, Xianying He, Dongqing Liu, Jinming Shi, Ming Ye, Linlin Wang, Yuntian Chu, Jie Zhao

**Affiliations:** 1 National Engineering Laboratory for Internet Medical Systems and Applications, The First Affiliated Hospital of Zhengzhou University, 1 Jianshe Road, Erqi District, Zhengzhou, 450052 Henan China; 2 Shanghai Artificial Intelligence Laboratory, Shanghai, China

**Keywords:** Telemedicine, Cancer treatment, Regional telemedicine service platform, Efficiency, Satisfaction

## Abstract

**Background:**

Telemedicine is beneficial for improving treatment efficiency and reducing medical expenses of cancer patients. This study focuses on cancer patients participating in teleconsultations through a regional telemedicine platform in China, analyzes the consultation process, and provides references for the clinical application of telemedicine.

**Methods:**

We collected information on teleconsultations of cancer patients conducted from 2015 to 2022 through the regional telemedicine platform. Utilizing SPSS 23.0 software, we conducted descriptive analysis to summarize the distribution of patient gender, age, region, and disease types. The ordinal logistic regression analysis was adopted to analyze the factors influencing the waiting time and consultation duration for teleconsultations.

**Results:**

From 2015 to 2022, a total of 23,060 teleconsultations were conducted for cancer patients via regional telemedicine platform, with an average growth rate of 11.09%. The main types of consultations were for lung cancer, liver cancer, and breast cancer, accounting for 18.14%, 10.49%, and 9.46% respectively. 57.05% of teleconsultations had a waiting time of less than 24 h, while patient age, consultation expert level, and disease type were the main factors influencing the waiting time. 50.06% of teleconsultations had a duration of more than 20 min, and the inviting hospital level and the title of invited consultant were the main factors influencing the consultation duration.

**Conclusions:**

In China, telemedicine has been widely employed in the clinical diagnosis and treatment of cancers, covering various types of oncological diseases. However, the waiting time for teleconsultations was generally more than 12 h, indicating the need to enhance consultation scheduling and allocate more expert resources to further optimize the efficiency of teleconsultations. Additionally, further exploration is required for remote health management of outpatients with cancers outside the hospital.

## Background

Telemedicine is a term coined in the 1970s, referring to treat patients from a distance. With the rapid development of telecommunications and IT technologies, the application of telemedicine has become more prevalent. Telemedicine usually refers to the use of modern communication technology, video technology, and information technology to achieve remote collection, transmission, storage, and exchange of patient information, aiming to provide diagnostic, monitoring, and treatment services to patients from a distance [1, 2]. In China, many remote areas lack high-quality medical resources [3], leading to the problem that patients often choose to seek treatment in urban areas or large hospitals, which not only increases the economic burden of patients, but also underutilizes medical resources. Telemedicine can overcome the geographical limitations of medical resources, thereby facilitating the sharing of high-quality medical resources to remote or rural areas, which plays an important role in enhancing primary medical service capabilities and reducing patient burden [1, 4]. After the outbreak of the COVID-19 pandemic in 2020, telemedicine has been widely used on a global scale [5]. With the help of telemedicine, the face-to-face contact between medical staff and patients has been reduced, thus reducing the risk of cross-infection and enabling patients in home isolation to receive effective treatment [6].

As telemedicine technology evolves and becomes more widely used, the types of services provided by telemedicine are constantly enriched, including teleconsultation, telepathology, teleradiology, tele-education and telemonitoring [7–9]. Among these services, teleconsultation is the most commonly used form in telemedicine. Teleconsultation refers to the medical consultation applied in advance by the inviter on telemedicine service platform and provided by the patient’s health condition discussion between the inviter and invitee through video conferencing equipment at a distance [10, 11]. To enhance diagnostic accuracy, teleconsultation is often supplemented by telepathology and teleradiology, where high-definition pathological and radiological images are uploaded to the telemedicine service platform, allowing doctors consult at any time and use as auxiliary diagnostic basis [12, 13].

With the popularization of telemedicine services, teleconsultation has been rapidly applied to various types of diseases for diagnosis and treatment. According to a survey conducted by Massachusetts General Hospital in the United States, 433 emergency departments of hospitals in the United States used telemedicine services for diagnosis and treatment of pediatric disease [14]. In 2020, the Boston Medical Center provided hybrid ophthalmology telemedicine therapy for 889 patients with eye diseases, and the results showed that the hybrid ophthalmology telemedicine treatment model for ophthalmology is an effective alternative to offline treatment [15]. The University of Malaya combined telemedicine services with smart glasses for intensive care in neurosurgery [16]. In China, Taizhou University integrated remote medical services with Internet of Things (IoT) technology for the treatment of type 2 diabetes, improving treatment efficiency [17]. The deep brain stimulation was combined with telemedicine to treat 196 patients with movement disorders and achieved a high patient satisfaction rate in Shanghai Ruijin Hospital [18]. In addition, several studies have shown that telemedicine played an important role in the diagnosis and treatment of mental disorders, diabetes, skin diseases, and cardiovascular diseases during the COVID-19 pandemic [19–21].

Currently, the world has achieved good results in the prevention and control of infectious diseases, with a continuous decrease in the incidence and mortality of infectious diseases. However, chronic diseases have gradually replaced infectious diseases as the main diseases affecting the health of Chinese population, and cancer is one of the main chronic diseases that affect the health of the population [22, 23]. The International Agency for Research on Cancer (IARC) released the latest global cancer burden data in 2020, assessing the burden and trends of cancer in 204 countries worldwide over the past decade. The number of global cancer deaths in 2019 rose to 10.0 million, and the number of new cases rose to 23.6 million. Compared with 2010, the deaths increased by 20.9% and the number of new cancer cases increased by 26.3% [24]. China has the highest incidence and mortality rates of cancer in the world. In 2016, there were 4.064 million new cases of cancer in China, and the incidence and mortality rates of cancer in China showed an increasing trend [25]. In 2017, lung cancer and liver cancer were among the top 10 diseases causing burden in China [26]. Overall, the burden of disease caused by cancers is constantly increasing and has been included in one of China’s five highly prioritized disease areas. The treatment and prevention of malignant cancers have become a focus of China’s health work.

In China, patients from rural areas account for nearly half of cancer patients, and the mortality rate of cancer patients in rural areas is higher than that in urban areas [25], which is related to the the shortage of high-quality medical resources in rural areas. Teleconsultation can effectively solve the problem that patients in rural areas cannot get effective treatment. Currently, telemedicine has been maturely applied in many specialized diseases, including the remote psychological consultation and telecare of cancer patients [27, 28]. However, existing research works mainly consist of review analysis and theoretical research and focus on tele-health management of cancer patients, and there has been little detailed research on the application process of the teleconsultation waiting time, teleconsultation duration and on the evaluation of the effectiveness of teleconsultations. The waiting time and duration for teleconsultations are crucial indicators for assessing consultation scheduling efficiency and outcomes. Analyzing waiting time and duration would contribute to exploring strategies for enhancing the quality of teleconsultation services. This study takes China’s largest regional telemedicine center as an example, collecting data from cancer cases consulted through this platform, conducting an in-depth analysis of the application process of teleconsultations among cancer patients, and takes the top ten malignancies in China’s mortality rate as an example [4], conducting an analysis of the deployment status of teleconsultation services, to provide reference for the application of telemedicine in cancer treatment.

## Methods

### Telemedicine service system and network construction

**Fig. 1:**
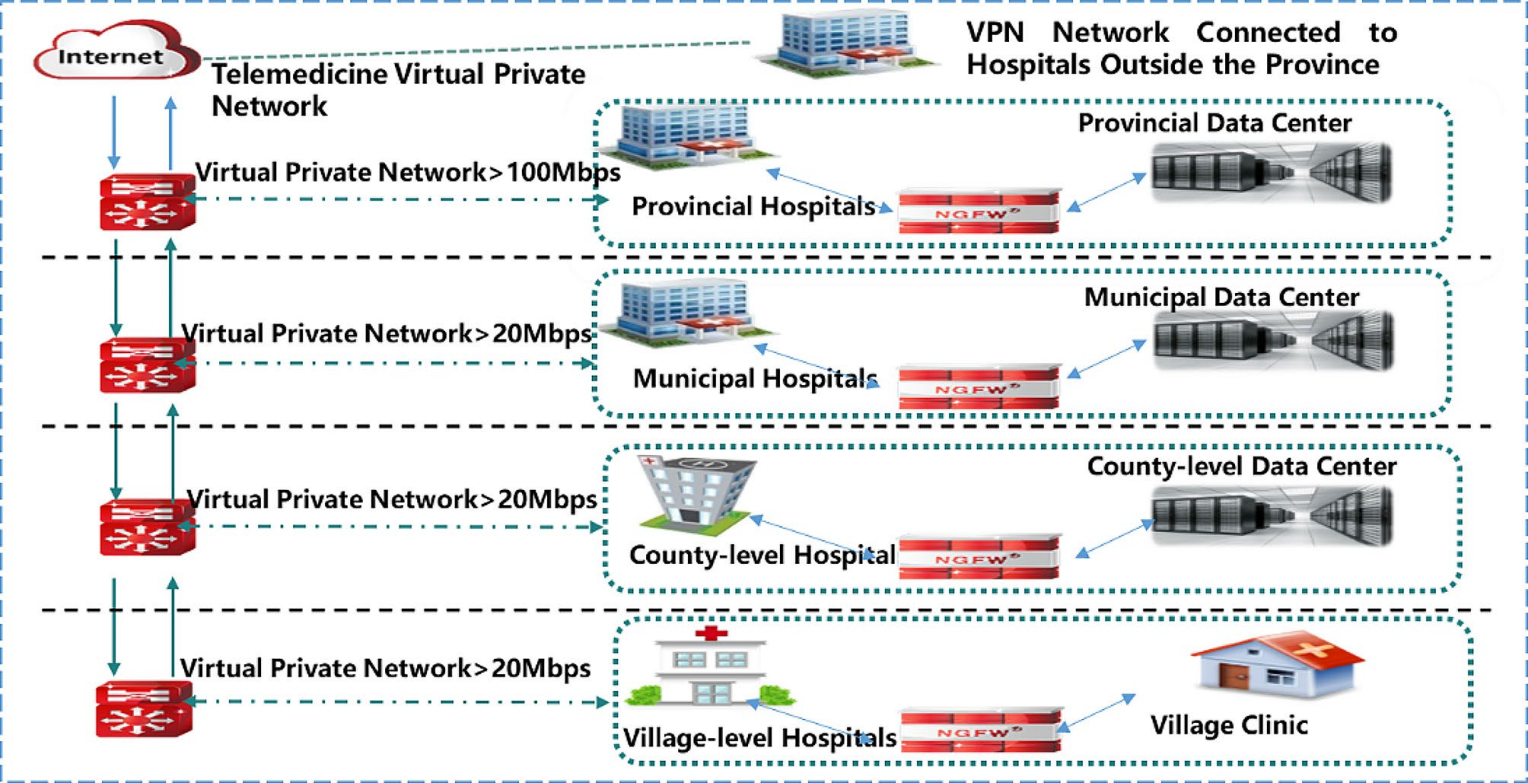
Telemedicine network connection of regional telemedicine center

To promote the development of telemedicine services among hospitals of different levels, Henan Province has entrusted the First Affiliated Hospital of Zhengzhou University, the largest hospital in Henan, to establish a regional telemedicine center, planning the configuration of telemedicine service facilities and network construction for hospitals of different levels in national wide. Large provincial hospitals were selected to establish provincial service centers; the largest municipal hospitals in 18 cities were selected to establish municipal service centers; and hospitals were selected in 108 counties to establish county-level service centers. All three levels of service centers, which operate on a “province-city-county” three-tier linkage, are managed and coordinated by the regional telemedicine center, facilitating cross-level telemedicine collaboration among hospitals in different regions. Meanwhile, virtual private network (VPN) with a bandwidth of no less than 100 megabits per second (Mbps) is used to connect hospitals at all levels, ensuring the security of medical information transmission. Telemedicine network connection of regional telemedicine center is shown in Fig. 1.

### Telemedicine service platform design and service process

**Fig. 2:**
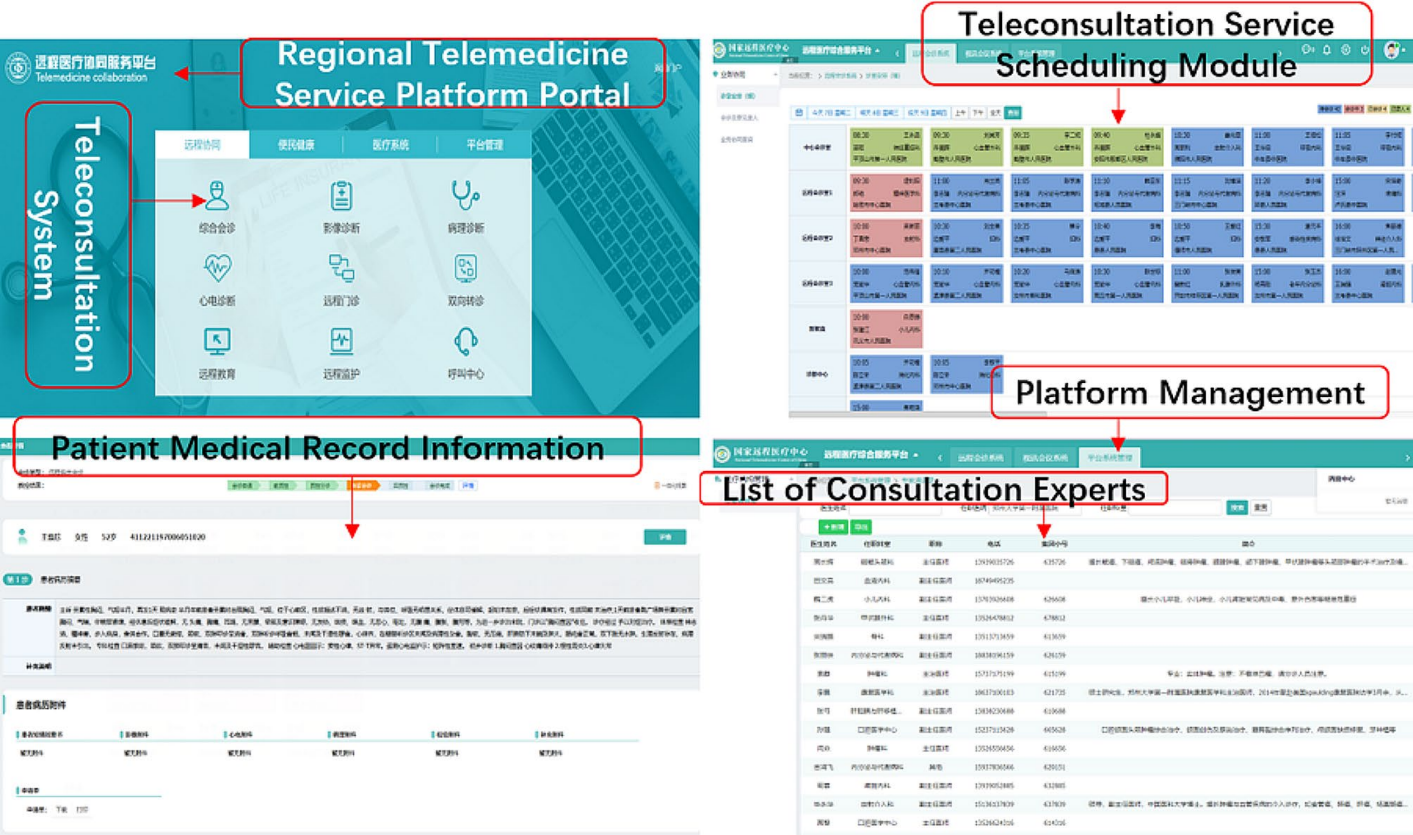
Telemedicine service platform portal and functional pages

The regional telemedicine service platform adopts a DevOps architecture, enabling rapid deployment and iterative updates of business systems compared to other remote healthcare platforms, reducing the costs of system development and deployment. The platform comprises four modules: teleconsultation, remote health management and follow-ups, medical information systems, and platform management, supporting various tele-healthcare services such as teleconsultations, remote disease diagnosis, and remote health management. In the telemedicine collaboration module, a teleconsultation platform is developed and deployed (see Fig. 2) to support the implementation of teleconsultation services. The platform has multiple functions, supporting the filling in and uploading of patient data, filling in the information of doctors applying for teleconsultation, as well as supporting doctors to select the department, expert, and teleconsultation time when applying for teleconsultation. The invited hospital can view the teleconsultation invitation information through the platform and fill in the arranged consultation time online. After the consultation time is confirmed, the platform will automatically send short messages to the inviting and invited doctors, and send reminder messages again before the consultation. Moreover, the invited doctor can fill in the diagnosis and treatment opinions and feedback to the inviting doctor after the implementation of teleconsultation. At present, the platform has registered 73 tertiary hospitals, 277 secondary hospitals, and 687 primary hospitals. The annual volume of teleconsultation services is at the forefront in China, achieving the scale and systemization of teleconsultation business.

**Fig. 3:**
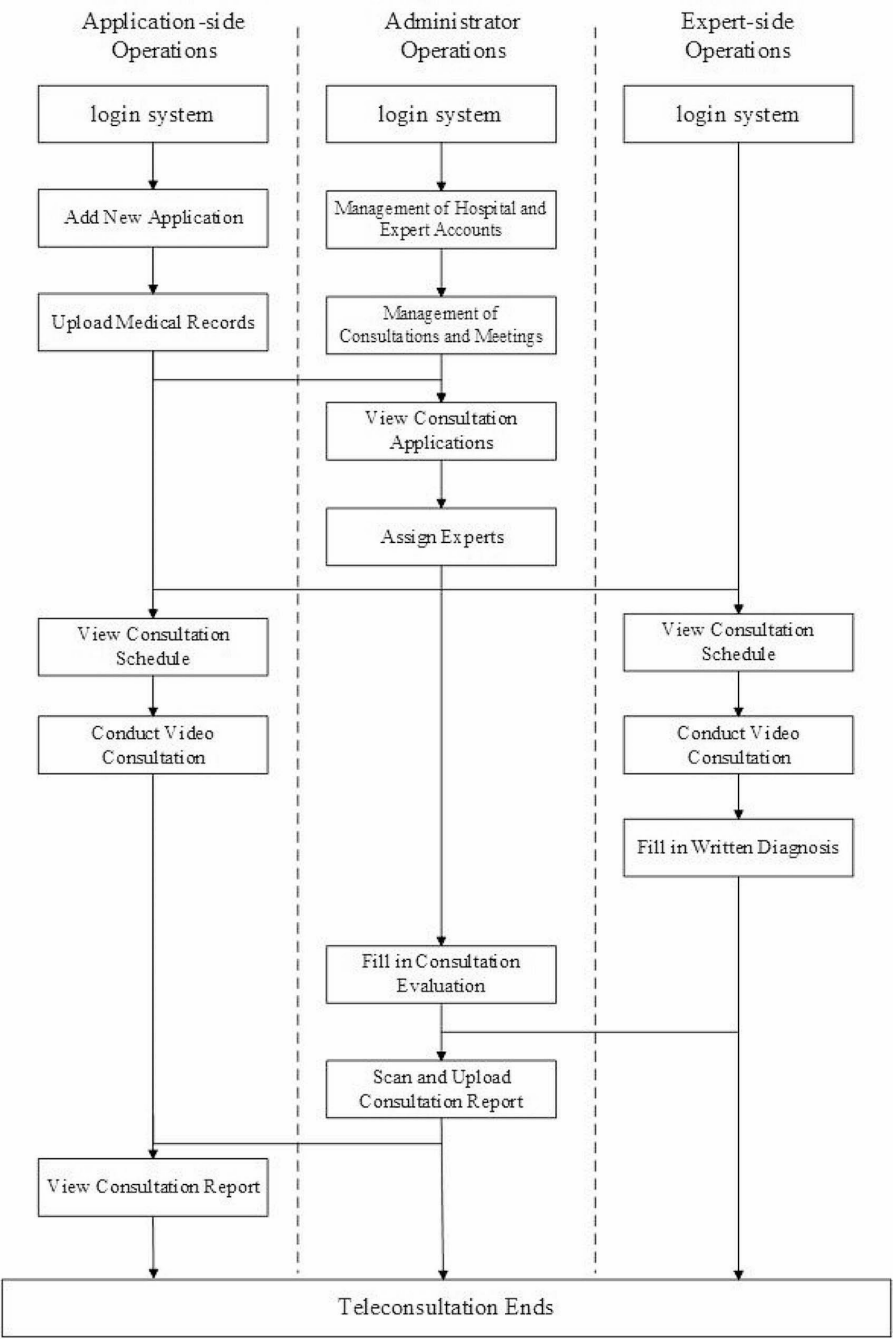
Teleconsultation service process

To ensure the standardized operation of teleconsultation service, we have formulated the teleconsultation service process. Firstly, the inviting doctor submits a teleconsultation request, fills in patient information and proposed teleconsultation information on the platform. After receiving the invitation, the invited hospital contacts the clinical doctor through the management personnel to determine whether they can participate in the teleconsultation and fills in the teleconsultation time on the platform. Then, inviting hospital and invited hospital conduct remote diagnosis and treatment communication through hardware video conference equipment. After the teleconsultation is completed, the invited doctor enters the consultation opinion, which is pushed to the inviting doctor by the platform. The specific teleconsultation process is shown in Fig. 3.

### Data collection

We collected data of all cancer cases that were consulted through the teleconsultation platform from January 2015 to December 2022. Our dataset contained patient basic information, information of inviting hospitals and doctors, information of invited doctors, waiting times, consultation durations, teleconsultation diagnosis and treatment suggestions, etc. The principal diagnosis of diseases was coded using ICD-10, and the teleconsultation of the top ten malignant cancers with high mortality rates in China, namely lung cancer, liver cancer, stomach cancer, colon and rectum cancer, esophageal cancer, pancreatic cancer, breast cancer, brain cancer, leukemia, and lymphoma, were analyzed. Other cases were classified as “other cancers”. The age distribution of patients was analyzed by grouping them into 0–14 years old, 15–24 years old, 25–34 years old, 35–44 years old, 45–54 years old, 55–64 years old, and ≥ 65 years old. The time difference between the teleconsultation implementation time and the teleconsultation application time was defined as the waiting time, and the time difference between the teleconsultation end time and the teleconsultation start time was defined as the duration time.

To analyze the effectiveness of teleconsultations, we utilized WJX (a survey software developed by a Chinese company) to create a questionnaire. Among the medical institutions participating in teleconsultations, we conducted an online survey, gathering responses from 976 inviting doctors involved in teleconsultations. We provided the questionnaire in the supplementary material. The questionnaire encompassed four parts: basic information about the doctors, details about their engagement in telemedicine services, their experience using telemedicine facilities, evaluations of the effectiveness and satisfaction with teleconsultations, as well as their willingness to continue using telemedicine services and suggestions for telemedicine. The questionnaire underwent reliability testing, showing a Cronbach’s α coefficient of 0.757. Validity testing indicated a KMO coefficient of 0.901 and Bartlett’s sphericity test with a *P* < 0.001, demonstrating good reliability and validity of the questionnaire.

### Statistical analysis

We used Excel (Microsoft Corp) software to establish a teleconsultation database for cancers, conducted structured processing and cleaning of the data. The line charts were used to describe the time trend of consultation cases, and pie charts were used to describe the distribution of teleconsultation waiting time and teleconsultation duration time. With the support of SPSS (version 23.0, IBM Corp) software, we conducted descriptive analysis of patient gender, age, geolocation, disease type, etc., using indicators such as quantity and composition ratio, and calculated the average annual growth rate of the indicators. Ordinal logistic regression analysis was used to analyze the impact of the rank of inviting hospitals, geolocation, patient status, referral recommendations and other factors on waiting time and duration of teleconsultations. Utilizing the survey data from the invited doctors participating in teleconsultations, we employed an ordinal regression model to analyze the factors influencing the effectiveness of teleconsultations and the satisfaction of the teleconsultations. In our statistical tests, the significance levels were set to *α* = 0.05.

## Results

### Analysis of trends in cancer teleconsultation volume

**Fig. 4:**
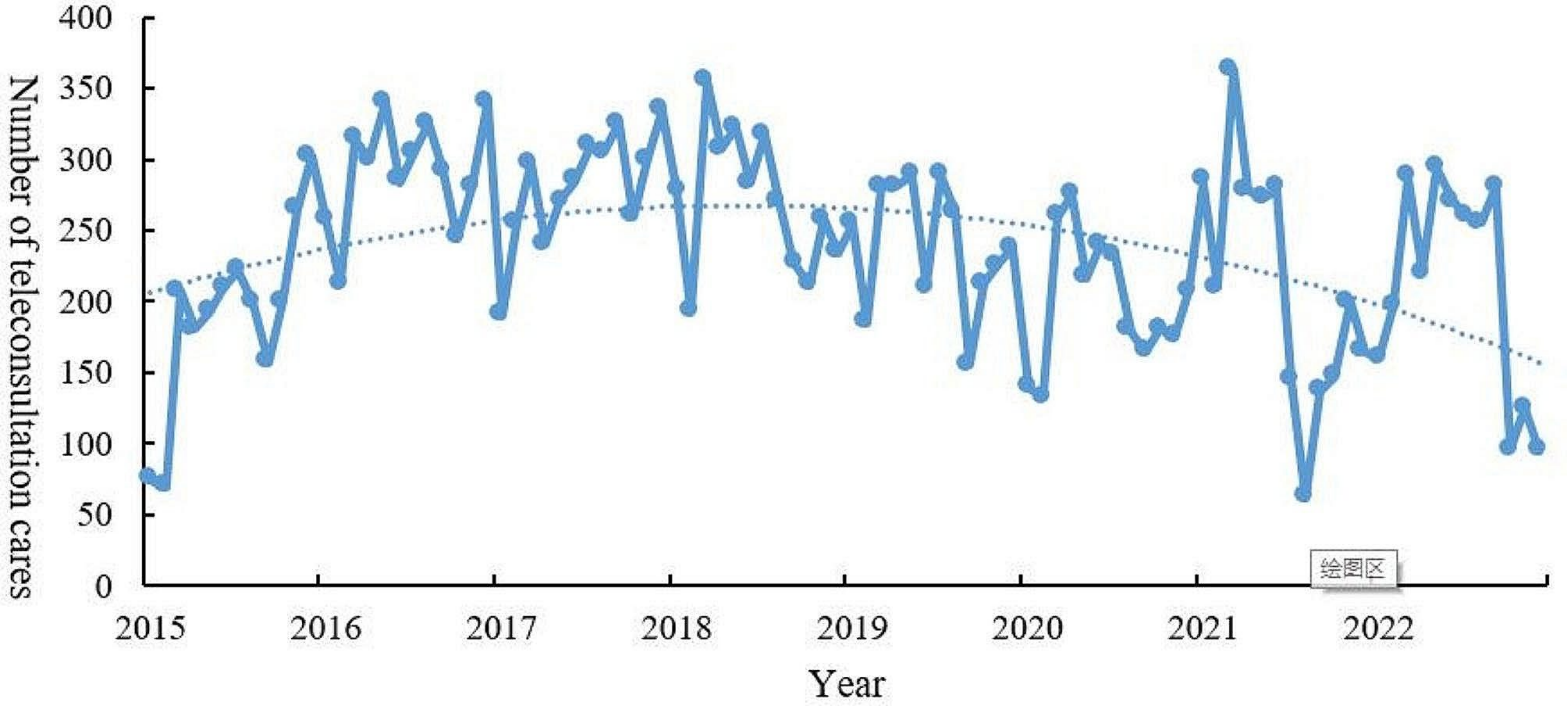
Trends in cancer teleconsultation cases from 2015 to 2022

From 2015 to 2022, a total of 23,060 cancer consultations were conducted through the regional telemedicine platform, and the overall consultation volume showed an increasing trend, with an average growth rate of 11.09%. The highest consultation volume was in 2016, with 3,581 cases, which represented a 54.49% increase compared to 2015. From 2017 to 2020, the annual consultation volume showed a downward trend, with the most significant decrease in 2020, which decreased by 16.17% (see Fig. 4).

### Basic information of cancer patients participating teleconsultation

**Fig. 5:**
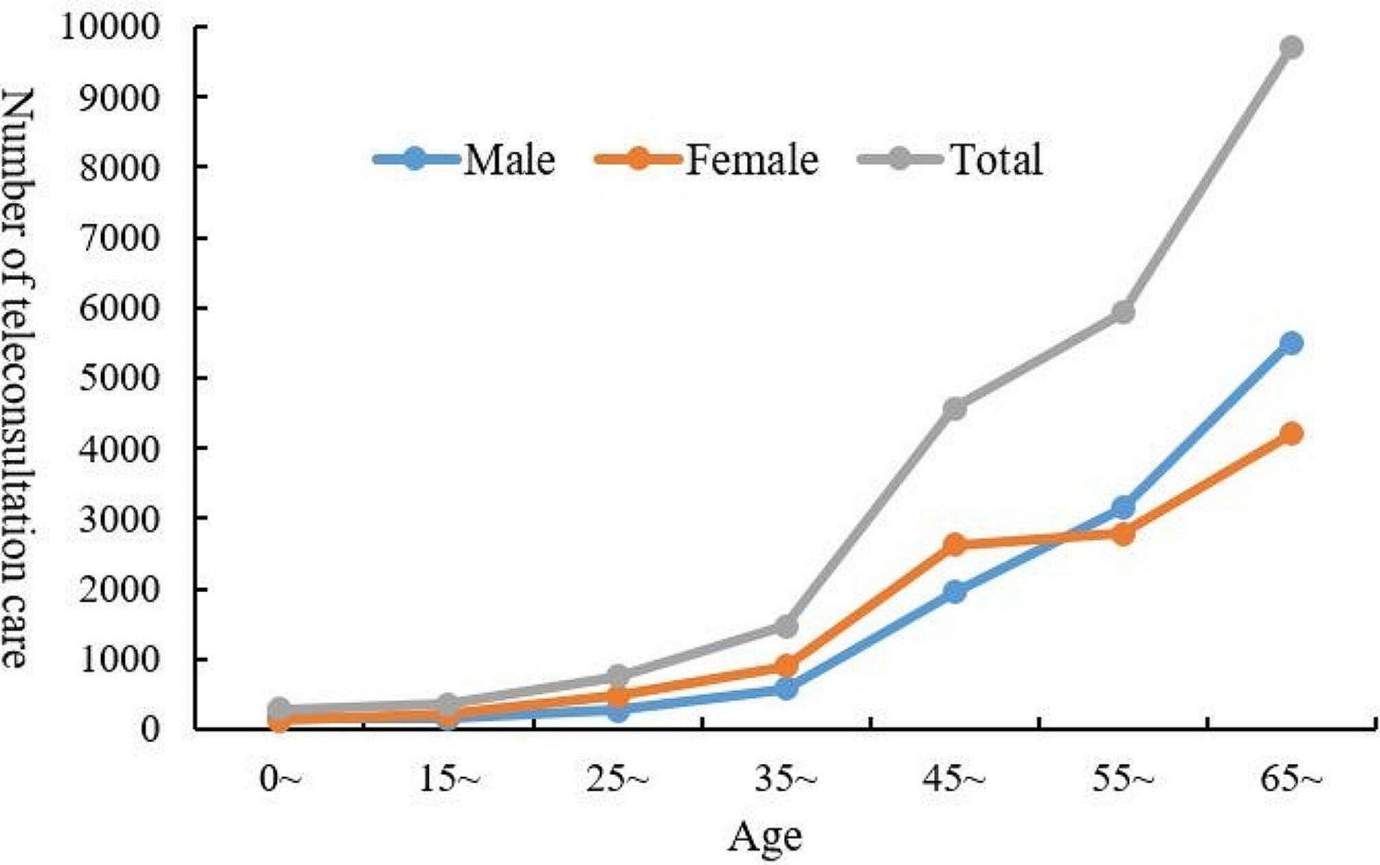
Distribution of age and gender among cancer consultation patients

**Fig. 6:**
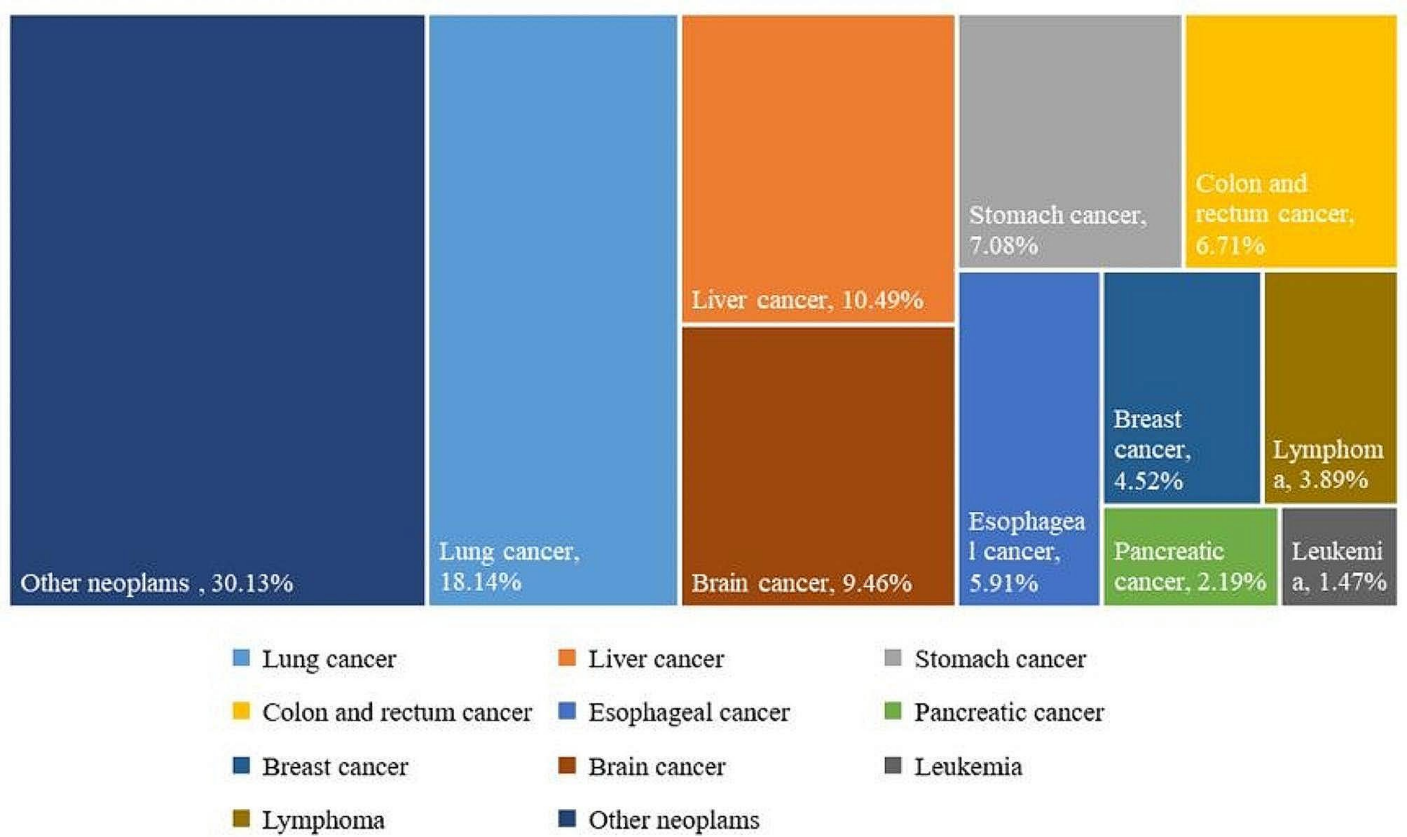
Types of diseases among cancer teleconsultation patients

**Fig. 7:**
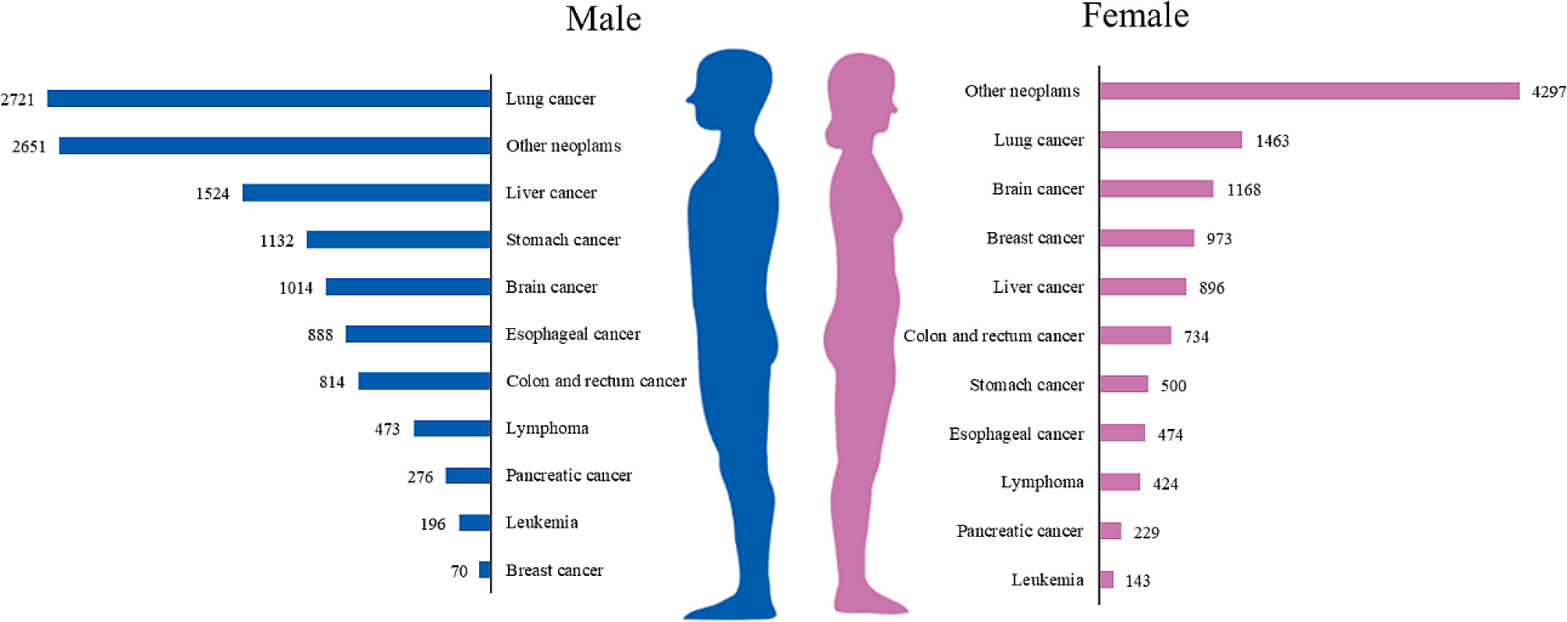
Disease distribution of cancer teleconsultation patients of different genders

Among the 23,060 cancer consultation patients, there were 11,759 male patients and 11,301 female patients, accounting for 50.99% and 49.01%, respectively. The age of the consultation patients was mainly distributed above 45 years old, with patients over 65 years old accounted for the highest proportion of 42.56%, while patients aged 45–55 and 55–65 accounted for 20.07% and 26.00%, respectively (see Fig. 5). The cancer patients who participated in teleconsultation mainly came from 22 cities in China, of which the patients from Henan Province accounted for 98.81%. We analyzed the distribution of cancer types among patients using teleconsultation, and the results showed that the number of lung cancer patients was the largest at 4,184, accounting for 18.14%, followed by liver cancer and breast cancer patients, accounting for 10.49% and 9.46%, respectively. Lymphoma and leukemia patients accounted for a relatively small proportion, at 3.89% and 1.47%, respectively. Other malignant cancer patients accounted for 30.13% (see Fig. 6). Male patients participating in consultations were mainly lung cancer, liver cancer, and gastric cancer, while female patients were mainly lung cancer, brain cancer, and breast cancer (see Fig. 7). From 2015 to 2022, breast cancer teleconsultation patients showed the fastest growth, with an increase of 44.94%, followed by colorectal cancer teleconsultation patients, with an increase of 39.39%, and liver cancer teleconsultation patients, with an increase of 36.72%, while Esophageal cancer teleconsultation patients and leukemia teleconsultation patients decreased by 27.97% and 26.67%, respectively (see Table 1).

**Table 1:** Distribution of diseases among teleconsultation patients

Diseases	2015	2016	2017	2018	2019	2020	2021	2022	Total	Change percent (%)
Lung cancer	431	667	638	614	457	466	381	530	4184	22.97
Liver cancer	256	398	353	360	290	223	190	350	2420	36.72
Stomach cancer	167	260	287	242	181	168	135	192	1632	14.97
Colon and rectum cancer	132	176	201	202	238	200	215	184	1548	39.39
Esophageal cancer	143	220	202	229	196	118	151	103	1362	-27.97
Pancreatic cancer	39	87	77	72	78	66	46	40	505	2.56
Breast cancer	89	142	157	152	135	110	129	129	1043	44.94
Brain cancer	185	313	336	306	322	225	256	239	2182	29.19
Leukemia	45	98	61	38	9	32	23	33	339	-26.67
Lymphoma	102	144	127	123	134	108	61	98	897	-3.92
Other neoplasm	729	1076	989	951	855	711	960	677	6948	-7.13

### The process of teleconsultation for cancer patients

The hospitals applying for teleconsultations were mainly secondary and tertiary hospitals, accounting for 84.11% and 15.89%, respectively. There are 1028 doctors, mainly with associate chief physician titles and chief physician titles, who have registered on the regional telemedicine platform. 50.47% of cancer patients receive consultations from chief physicians, 49.17% from associate chief physicians, and only 0.36% of cancer patients receive consultations from attending doctors.

**Fig. 8:**
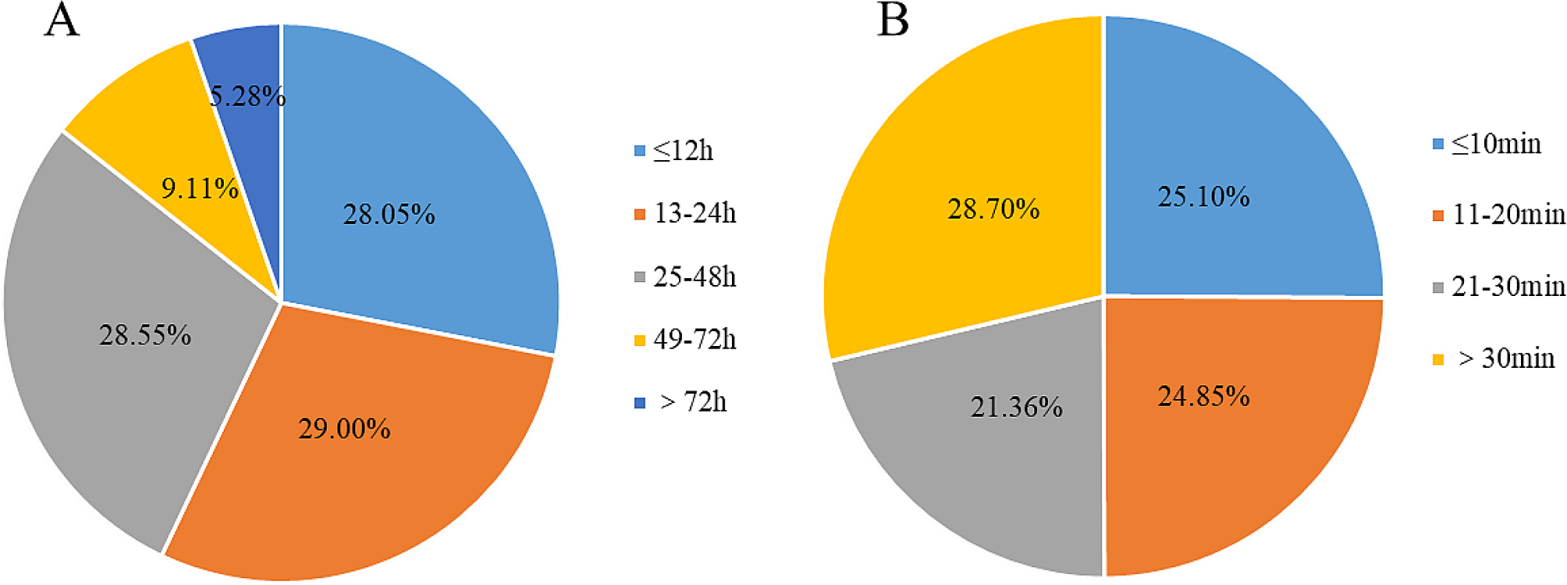
Time distribution for waiting and process duration of teleconsultations for cancer patients.: (**A**) Distribution of waiting time for teleconsultations; (**B**) Distribution of duration for teleconsultations

This study analyzed the consultation waiting time of 22,957 cancer patients using teleconsultation, and the results showed that most consultation waiting times did not exceed 48 h, 28.05% of consultations waiting did not exceed 12 h, 29.00% of consultations waiting time did not exceed 13–24 h, and 28.55% of consultations time did not exceed 25–48 h. At the same time, we analyzed the teleconsultation duration for 11,635 cancer patients, which was mainly divided into ≤ 10 min group, 11–20 min group, 21–30 min group, and more than 30 min group. The cases with consultation times exceeding 30 min accounted for the highest proportion at 28.70% (see Fig. 8). Through teleconsultations, 12.66% of patients were recommended to seek treatment at larger hospitals.

### Analysis of factors affecting teleconsultations for cancer patients

Using patient basic information, the level of the hospital applying for consultation, the title of invited consultant, disease category, etc. as independent variables, an ordered logistic regression model was used to analyze the factors influencing the waiting time for teleconsultations in cancer patients. The results showed that the year of consultation application, region, patient age, and disease category were the main factors affecting the waiting time for consultations. Compared to 2022, the waiting time for consultations was longer in 2018–2021, with β = 0.445, 0.229, 0.198, and 0.254 respectively (*P* < 0.05). Patients from Henan Province had longer waiting times, with β = 0.358 (*P* = 0.018). Compared to other cancer patients, brain cancer and stomach cancer patients had shorter waiting times, while breast cancer and lymphoma patients had longer waiting times (see Table 2).

**Table 2:** Ordinal logistic regression analysis results for waiting time of teleconsultations

Variable	Coefficients (95%CI)	S.E.	Wald	*P*
Year (ref = 2022)				
2015^*^	-1.149(-1.310, -0.988)	0.082	196.179	<0.001
2016^*^	-0.464(-0.616,-0.313)	0.077	36.002	<0.001
2017	0.125(-0.027,0.277)	0.078	2.618	0.106
2018^*^	0.445(0.292,0.598)	0.078	32.528	<0.001
2019^*^	0.229(0.069,0.389)	0.082	7.836	0.005
2020^*^	0.198(0.034,0.361)	0.083	5.613	0.018
2021^*^	0.254(0.082,0.427)	0.088	8.332	0.004
Region (ref = other provinces)				
Henan Province^*^	0.358(0.061,0.654)	0.151	5.584	0.018
Applicant hospital level (ref = Second-class and below hospital)				
Tertiary hospital	-0.022(-0.101,0.057)	0.040	0.300	0.584
Sex (ref = Female)				
Male	0.016(-0.04,0.073)	0.029	0.319	0.572
Age (ref = Over 65 years old)				
0–14 years old	-0.015(-0.285,0.256)	0.138	0.011	0.916
15–24 years old	-0.186(-0.419,0.047)	0.119	2.438	0.118
25–34 years old^*^	-0.193(-0.358,-0.029)	0.084	5.315	0.021
35–44 years old	-0.099(-0.216,0.017)	0.060	2.789	0.095
45–54 years old^*^	-0.095(-0.169,-0.021)	0.038	6.264	0.012
55–64 years old^*^	-0.071(-0.138,-0.005)	0.034	4.462	0.035
Title of invited consultant (ref = Chief physician)				
Attending doctor	0.554(-0.013,1.122)	0.290	3.666	0.056
Associate chief physician	0.03(-0.024,0.085)	0.028	1.201	0.273
Diseases (ref = Other neoplasm )				
Lung cancer	0.026(-0.056,0.107)	0.042	0.383	0.536
Liver cancer	-0.08(-0.178,0.017)	0.050	2.595	0.107
Stomach cancer^*^	-0.142(-0.253,-0.031)	0.057	6.286	0.012
Colon and rectum cancer	0.005(-0.107,0.117)	0.057	0.008	0.929
Esophageal cancer	-0.024(-0.141,0.094)	0.060	0.155	0.694
Pancreatic cancer	-0.049(-0.24,0.142)	0.097	0.251	0.617
Breast cancer^*^	0.24(0.108,0.372)	0.067	12.731	0.000
Brain cancer^*^	-0.265(-0.379,-0.15)	0.058	20.494	0.000
Leukemia	0.085(-0.135,0.306)	0.112	0.578	0.447
Lymphoma^*^	0.179(0.03,0.327)	0.076	5.581	0.018
Transfer treatment (ref = No)				
Yes	0.013(-0.069,0.095)	0.042	0.094	0.759

* Significant variable with *P*<0.05

Furthermore, we analyzed the impact of patient conditions, hospital application conditions, and waiting time on the duration of teleconsultations for cancer patients. The results demonstrated that compared to 2022, the consultation duration was shorter in 2015–2019, with β = -1.051, -1.652, -1.146, -0.565 and − 0.585 respectively (*P* < 0.001). When the applying hospital was a tertiary hospital, the consultation duration was longer (β = 0.256, *P* < 0.001). Experts with associate chief physician title had longer consultation durations compared those with chief physician title, (β = 0.124, *P* = 0.002). Patients with brain cancer, leukemia, and lymphoma had longer consultation durations compared with other cancer patients, with β = 0.179, 0.519, and 0.348 respectively (*P* < 0.05) (see Table 3).

**Table 3:** Ordinal logistic regression analysis results for the duration of teleconsultations

Variable	Coefficients (95%CI)	S.E.	Wald	*P*
Year (ref = 2022)				
2015^*^	-1.051(-1.223, -0.880)	0.088	144.008	<0.001
2016^*^	-1.652(-1.816, -1.488)	0.084	388.372	<0.001
2017^*^	-1.146(-1.318, -0.975)	0.088	170.923	<0.001
2018^*^	-0.565(-0.755, -0.375)	0.097	34.084	<0.001
2019^*^	-0.585(-0.796, -0.373)	0.108	29.403	<0.001
2020	-0.136(-0.368, 0.097)	0.119	1.304	0.253
2021	0.024(-0.193, 0.241)	0.111	0.047	0.828
Region (ref = other provinces)				
Henan Province	0.201(-0.230, 0.633)	0.220	0.835	0.361
Applicant hospital level (ref = Second-class and below hospital)				
Tertiary hospital^*^	0.256(0.135, 0.377)	0.062	17.291	<0.001
Sex (ref = Female)				
Male	0.006(-0.075, 0.088)	0.042	0.022	0.882
Age (ref = Over 65 years old)				
0–14 years old	-0.137(-0.504, 0.230)1	0.187	0.537	0.464
15–24 years old	-0.064(-0.372, 0.245)	0.157	0.164	0.686
25–34 years old	0.159(-0.077, 0.395)	0.121	1.736	0.188
35–44 years old	-0.029(-0.194, 0.136)	0.084	0.120	0.729
45–54 years old	0.002(-0.104, 0.108)	0.054	0.002	0.966
55–64 years old	0.047(-0.050, 0.143)	0.049	0.891	0.345
Title of invited consultant (ref = Chief physician)				
Attending doctor	0.086(-0.732, 0.905)	0.418	0.043	0.836
Associate chief physician^*^	0.124(0.045, 0.203)	0.040	9.533	0.002
Diseases (ref = Other neoplasm )				
Lung cancer	0.104(-0.015, 0.222)	0.060	2.949	0.086
Liver cancer	0.078(-0.059, 0.214)	0.070	1.238	0.266
Stomach cancer	0.112(-0.052, 0.276)	0.084	1.797	0.180
Colon and rectum cancer	-0.053(-0.217, 0.110)	0.083	0.411	0.521
Esophageal cancer	-0.088(-0.263, 0.086)	0.089	0.987	0.320
Pancreatic cancer	0.110(-0.166, 0.387)	0.141	0.611	0.434
Breast cancer	0.050(-0.141, 0.241)	0.098	0.262	0.609
Brain cancer^*^	0.179(0.019, 0.338)	0.081	4.801	0.028
Leukemia^*^	0.519(0.222, 0.816)	0.151	11.738	0.001
Lymphoma^*^	0.348(0.130, 0.566)	0.111	9.783	0.002
Waiting time	0.009(-0.027, 0.045)	0.018	0.238	0.626
Transfer treatment (ref = No)				
Yes	0.053(-0.079, 0.185)	0.067	0.608	0.436

* Significant variable with *P*<0.05

### Teleconsultation effectiveness and satisfaction evaluation

According to the evaluation of the inviting doctors, 3.89% of doctors believe that the diagnosis results of teleconsultations are inconsistent with the original diagnosis, 71.21% of doctors believe that partial results are consistent, and 24.90% of doctors believe that the results are completely consistent. We used an ordinal regression model to analyze the factors affecting the effectiveness of teleconsultations. The results showed that the use of video conference terminals and service processes were the main factors affecting the consultation results. The higher the satisfaction with the use of video conference terminals and service processes, the better the consultation results, with β values of 0.717 and 0.564, respectively (*P* < 0.05). The doctors’ education level, professional title, and years of experience in telemedicine were not related to the consultation results (see Table 4).

**Table 4:** Ordinal logistic regression analysis results for the effectiveness of teleconsultations

Variable	Coefficients (95%CI)	S.E.	Wald	*P*
Title of invited consultant (ref = Primary title)				
Middle title	0.278(-0.147,0.704)	0.217	1.641	0.200
High title	0.080(-0.33,0.49)	0.209	0.147	0.701
Education (ref = Doctoral degree)				
High school and below Associate degree	1.202(-0.123,2.527)	0.676	3.160	0.075
Bachelor degree	0.809(-0.446,2.065)	0.641	1.595	0.207
Master degree	0.758(-0.538,2.054)	0.661	1.315	0.252
Years of working in telemedicine (ref = Over 10 years)				
less than 1 year	-0.152(-1.015,0.712)	0.440	0.118	0.731
2–4 years	-0.347(-1.158,0.463)	0.414	0.705	0.401
5–10 years	-0.35(-1.251,0.55)	0.460	0.582	0.446
Satisfaction degree toward hardware equipment	0.419(-0.017,0.856)	0.223	3.552	0.059
Satisfaction degree toward audio and video clarity^*^	0.717(0.317,1.118)	0.204	12.329	<0.001
Satisfaction degree toward service process^*^	0.564(0.042,1.085)	0.266	4.489	0.034
Satisfaction degree toward service environment	0.462(-0.072,0.995)	0.272	2.876	0.090
During time	0.076(-0.078,0.231)	0.079	0.936	0.333

* Significant variable with *P*<0.05

We collected evaluations from inviting doctors on the overall satisfaction of teleconsultations and found that 95.29% of doctors expressed satisfaction with teleconsultations. Using an ordinal regression model, we analyzed the impact of doctors’ education level, professional title, consultation duration, waiting time, satisfaction with the use of video conference terminal devices, and other variables on teleconsultation satisfaction. We found that the convenience of device operation, satisfaction with the use of video conference terminals, satisfaction with the service process, and satisfaction with the consultation results were all factors influencing the overall satisfaction of teleconsultations, with β values of 0.827, 0.732, 0.970, and 1.855, respectively (*P* < 0.001). Implementing incentive measures to encourage doctors to participate in teleconsultations will improve satisfaction (β = 1.003, *P* < 0.001) (see Table 5).

**Table 5:** Ordinal logistic regression analysis results for the satisfaction of teleconsultations

Variable	Coefficients (95%CI)	S.E.	Wald	*P*
Sex (ref = Female)				
Male	0.027(-0.352,0.391)	0.188	0.021	0.918
Age (ref = Over 55 years old)				
0–14 years old	0.788(-6.261,7.838)	3.597	0.048	0.827
15–24 years old	-0.951(-3.209,1.306)	1.152	0.682	0.409
25–34 years old	-0.740(-2.749,1.269)	1.025	0.522	0.470
35–44 years old	-0.550(-2.503,1.404)	0.997	0.304	0.581
45–54 years old	-0.669(-2.613,1.275)	0.992	0.455	0.500
Title of invited consultant (ref = Primary title)				
Middle title	-0.005(-0.719,0.709)	0.364	0.000	0.989
High title	-0.112(-0.697,0.473)	0.298	0.140	0.708
Education (ref = Doctoral degree)				
High school and below Associate degree	0.841(-0.71,2.392)	0.791	1.129	0.288
Bachelor degree	0.430(-0.988,1.848)	0.723	0.353	0.552
Master degree	0.231(-1.231,1.694)	0.746	0.096	0.757
Years of working in telemedicine (ref = Over 10 years)				
less than 1 year	-0.845(-2.196,0.507)	0.690	1.500	0.221
2–4 years	-0.759(-2.054,0.535)	0.660	1.323	0.250
5–10 years	-0.919(-2.303,0.465)	0.706	1.695	0.193
Informatization level meets the demand of telemedicine (ref = Yes)				
No	-0.239(-1.061,0.582)	0.419	0.327	0.568
Use convenience of hardware equipment^*^	0.827(0.421,1.233)	0.207	15.930	<0.001
Satisfaction degree toward audio and video clarity^*^	0.732(0.364,1.099)	0.187	15.255	<0.001
Satisfaction degree toward service process^*^	0.970(0.441,1.499)	0.270	12.911	<0.001
Satisfaction degree toward service environment	0.481(-0.042,1.004)	0.267	3.244	0.072
Satisfaction degree toward teleconsultation result^*^	1.855(1.455,2.256)	0.204	82.570	<0.001
Stimulate measure(ref = No)				
Yes^*^	1.003(0.586,1.42)	0.213	22.222	<0.001
Waiting time	0.078(-0.12,0.275)	0.101	0.595	0.441
During time	0.063(-0.135,0.261)	0.101	0.390	0.532

* Significant variable with *P*<0.05

## Discussion

Telemedicine has advantages in enhancing the service capabilities of primary medical institutions, improving the convenience of medical treatment, and reducing the economic burden of diagnosis and treatment for patients. As the global disease burden of cancer is constantly growing [29], applying telemedicine to the treatment of cancer patients can help improve the treatment level of cancer patients in rural and remote areas and reduce the economic burden of cancer. Taking the largest regional telemedicine center in China as an example, this study provided a detailed analysis of 23,060 cases of cancer teleconsultation conducted by the regional telemedicine center from 2015 to 2022. It included the disease distribution of cancer patients, consultation process, consultation results, etc., and analyzed the factors affecting the efficiency of cancer teleconsultation, so as to provide insights for the application of telemedicine in cancer diagnosis and treatment.

Longitudinal analysis revealed that from 2015 to 2022, the number of cancer patients participating in teleconsultation showed an overall increasing trend, with an average growth rate of 11.09%, indicating that the application scope of telemedicine in cancer is gradually expanding. In recent years, with the promotion and application of telemedicine in the worldwide, it has been widely used in diabetes, otolaryngology, pediatrics, and other fields [30–32]. The rapid advancement of telemedicine technology has evolved from early consultations conducted through video conferencing terminals to consultations via software-based video conferencing systems. This progression has consistently reduced costs and improved convenience, expanding the scope of telemedicine applications [33, 34]. In the future, the development of teleconsultation mobile application could enable doctors to offer remote diagnosis and treatment services to patients anytime and anywhere, leveraging the value of telemedicine in cancer care. The geographical coverage of its application has also been expanding, which is consistent with the results of this study. Along with the growth in the volume of cancer teleconsultations, we observed a decline in consultation volume in 2018–2019. The year 2020 was a period of high incidence of the COVID-19 pandemic, with many doctors involved in epidemic prevention and control, resulting in a decrease in the number of doctors participating in teleconsultations.

Lung cancer, liver cancer and stomach cancer patients were the majority of cancer patients participating in teleconsultation, and the annual consultation volume showed an increasing trend, which was related to the high incidence of lung cancer, liver cancer, and stomach cancer in China [25]. Brain cancer consultations ranked third in terms of volume. Although the incidence of brain cancer was lower than that of colorectal cancer and breast cancer, the treatment of brain cancer mainly relied on surgical resection [35], which required a high level of surgical skills. Primary healthcare institutions usually do not possess the capability to perform complex brain surgeries and therefore require remote guidance from experts in higher-level hospitals. Thus, the volume of brain cancer consultations was relatively high. The age of Chinese cancer patients was mainly above 40 years old, with the peak incidence occurring between 60 and 79 years old. Therefore, the age distribution of cancer patients participating in teleconsultations was mainly above 45 years old, and the number of patients participating in teleconsultations increases with age. In China and even globally, cancer remains a primary factor affecting public health, with long treatment cycles and requiring extensive rehabilitation and follow-ups. Telemedicine offers advantages such as convenience and real-time assistance. Apart from conducting teleconsultations for cancer patients, it is recommended to integrate telemedicine across the entire lifecycle of cancer patients’ health management. This includes remote screening for high-risk groups, utilizing wearable devices for remote health monitoring of cancer patients to promptly alert about potential risks, providing remote surgical guidance for patients, and offering remote follow-ups and rehabilitation guidance for discharged cancer patients. Currently, telemedicine’s application in cancer treatment primarily focuses on teleconsultations, while its potential in cancer prevention and control awaits further exploration.

Through analyzing the information of applying hospitals for cancer teleconsultations, it was found that the volume of teleconsultation requests from secondary hospitals is much higher than that from tertiary hospitals. Secondary hospitals mainly refer to county-level medical institutions with lower medical capabilities and limited access to high-quality medical resources in their regions, resulting in a higher demand for telemedicine services. Meanwhile, the regional telemedicine center introduced in this study mainly assigned experts with chief physician titles to participate in teleconsultations to ensure the effectiveness. As a result, only 12.66% of patients were recommended for referral to higher-level hospitals for treatment after consultation. This proves that telemedicine can significantly enhances the capability of primary healthcare institutions in treating cancer patients. The number of cancer patients in China ranks among the highest globally. Large medical institutions are overwhelmed with an excess of cancer patients, exceeding doctors’ capacities. Telemedicine offers a new model for treating cancer patients. We believe that a majority of postoperative recovery and chemotherapy-phase cancer patients can be admitted to primary healthcare institutions and treated according to the treatment plans formulated remotely by experts from higher-level hospitals. This approach not only alleviates the financial burden on patients but also rationalizes the utilization of medical resources. Currently, China is using telemedicine as a link to establish a multi-institutional collaboration in oncology, effectively enhancing the diagnostic and treatment capabilities of primary healthcare institutions and reducing the rate of referrals to higher-level hospitals [36].

The waiting time for teleconsultation is an important factor affecting the efficiency of telemedicine service. We analyzed the waiting time for cancer teleconsultations and found that 28.05% of hospitals received consultation arrangements within 12 h after submitting teleconsultation requests, while 42.95% of consultations had waiting times exceeding 24 h. Multifactor analysis revealed that compared to 2022, the waiting time for consultations was shorter in 2015–2016, but increased from 2018 to 2021. As telemedicine is being promoted, the annual volume of consultations is gradually increasing. However, the efficiency of scheduling consultations may decrease, leading to longer waiting times. Among the patients applying for teleconsultations, there are a higher number from Henan province, resulting in longer waiting times compared to patients from other regions. The efficiency of teleconsultation services primarily depends on the number of invited consultants participating in the consultations. Considering these aspects, we suggest that higher-level medical institutions formulate incentive measures, increase the participation of invited experts in teleconsultations, diversify the expertise of consulting doctors, thereby ensuring that patients from different regions and with different conditions receive timely remote treatment. Additionally, we recommend further promoting “province-city-county” three-tier linkage model for telemedicine services. Primary healthcare institutions should first apply for teleconsultations from the higher-tier medical institutions. If the issues cannot be resolved, then they can seek support from even higher-level hospitals, aiming to prevent a concentration of consultation requests from primary healthcare institutions to higher-level medical institutions, consequently reducing the waiting time for consultations.

This study found that 71.30% of cancer teleconsultations lasted within 30 min, with some consultations even lasting less than 10 min. The duration of teleconsultations affects the accuracy of patient case data exchange and the effectiveness of consultations. Given the heavy workload of clinical doctors, many tend to select shorter time slots for consultations, especially senior experts, whose participation duration is even shorter. To enhance the effectiveness of consultations, it’s recommended for hospitals to establish a scheduling system for teleconsultations, with departments coordinating doctors and consultation times, ensuring adequate time for doctors’ participation. Additionally, a teleconsultation mobile app could be developed, allowing doctors to participate in teleconsultations anytime and anywhere, thereby increasing their participation duration in consultations. Furthermore, we found that the convenience of operating equipment, the effectiveness of audiovisual terminals, and service procedures are key factors affecting the satisfaction and effectiveness of consultations. Currently, while China has set unified technical requirements for telemedicine equipment and information systems, there’s no standardized process for telemedicine services, nor is there performance evaluation or supervision of these services. To enhance the value of telemedicine in cancer treatment, efforts should focus on advancing the development of teleconsultation audiovisual transmission equipment, improving information collection and transmission efficiency. More importantly, the crucial aspect lies in accelerating the establishment of standardized protocols for service processes, strengthening supervision and evaluation of telemedicine services and enhancing telemedicine service quality.

### Limitations

This study comprehensively analyzed the application and service efficiency of teleconsultations in cancer patients, providing valuable references for the implementation of teleconsultations for cancers in other countries and regions. Telemedicine involves multiple participants, including inviting and invited medical institutions, inviting and invited doctors, as well as patients. Due to the complexity of surveying the participants, this study did not investigate and analyze their opinions on teleconsultation from the perspectives of patients and doctors, which will be the focus of our next study. We will take into account the opinions of different participants in teleconsultations and summarize recommendations to improve the application effectiveness of teleconsultations for cancer diseases.

## Conclusions

In this paper, we analyzed the teleconsultation services for cancer patients conducted through the largest regional telemedicine platform in China from 2015 to 2022. The findings showed an increasing trend in the application of teleconsultations for cancer diseases. Secondary hospitals were the main applicants for consultations, and the participating patients were mainly those with lung cancer, liver cancer, brain cancer, and stomach cancer. The age distribution of patients was predominantly above 45 years old, and the suggested referral rate after consultations was 12.66%. The level and region of the applying hospital, the level of invited consultant, and the type of disease consulted were identified as the main factors influencing the efficiency and effectiveness of teleconsultation. To improve the efficiency of teleconsultation services for cancer patients, it is necessary to increase the allocation of expert resources, particularly in the fields of breast cancer and lymphoma, optimize the scheduling process and management system of teleconsultation, reduce waiting time for consultations, ensure reasonable consultation duration, and provide scientific and reasonable diagnosis and treatment recommendations for primary healthcare institutions.

## Data Availability

The datasets used and/or analysed during the current study available from the corresponding author on reasonable request.
